# Anti-Angiogenic Therapy Induces Integrin-Linked Kinase 1 Up-Regulation in a Mouse Model of Glioblastoma

**DOI:** 10.1371/journal.pone.0013710

**Published:** 2010-10-29

**Authors:** Chiara Verpelli, Giulio Bertani, Valentina Cea, Monica Patti, Andreas Bikfalvi, Lorenzo Bello, Carlo Sala

**Affiliations:** 1 ELAT (European Laboratory for Angiogenesis and Translational Research), University of Milan, Milan, Italy; 2 CNR Neuroscience Institute and Department of Pharmacology, University of Milan, Milan, Italy; 3 INSERM U920 (ex E0113) and University of Bordeaux 1, Talence, France; 4 Neurosurgery, Department of Neurological Sciences, University of Milan, Milan, Italy; Dana-Farber Cancer Institute, United States of America

## Abstract

**Background:**

In order to improve our understanding of the molecular pathways that mediate tumor proliferation and angiogenesis, and to evaluate the biological response to anti-angiogenic therapy, we analyzed the changes in the protein profile of glioblastoma in response to treatment with recombinant human Platelet Factor 4-DLR mutated protein (PF4-DLR), an inhibitor of angiogenesis.

**Methodology/Principal Findings:**

U87-derived experimental glioblastomas were grown in the brain of xenografted nude mice, treated with PF4-DLR, and processed for proteomic analysis. More than fifty proteins were differentially expressed in response to PF4-DLR treatment. Among them, integrin-linked kinase 1 (ILK1) signaling pathway was first down-regulated but then up-regulated after treatment for prolonged period. The activity of PF4-DLR can be increased by simultaneously treating mice orthotopically implanted with glioblastomas, with ILK1-specific siRNA. As ILK1 is related to malignant progression and a poor prognosis in various types of tumors, we measured ILK1 expression in human glioblatomas, astrocytomas and oligodendrogliomas, and found that it varied widely; however, a high level of ILK1 expression was correlated to a poor prognosis.

**Conclusions/Significance:**

Our results suggest that identifying the molecular pathways induced by anti-angiogenic therapies may help the development of combinaatorial treatment strategies that increase the therapeutic efficacy of angiogenesis inhibitors by association with specific agents that disrupt signaling in tumor cells.

## Introduction

The dependence of tumor growth and metastasis on angiogenesis provides a powerful rationale for anti-angiogenic approaches for the treatment of glioblastoma and other solid tumors. Targeting blood vessels in brain tumors is a particularly attractive strategy, given their characteristic high degree of endothelial proliferation, vascular permeability, and expression of pro-angiogenic growth factors, [e.g. vascular endothelial growth factor (VEGF)] [1], [2], [3], [4].

In the case of glioblastomas, anti-angiogenic agents have been used in combination with chemotherapy but, after a certain amount of time, tumor growth resumes. It has recently been suggested that cells evade anti-angiogenic therapies by up-regulating alternative signaling circuits [5]. It is therefore useful to identify pathways that are associated with tumor angiogenesis and the response to anti-angiogenic therapy. This may help in developing new specific combinatorial therapeutic strategies.

We used a proteomic approach to investigate the “proteome response” to the treatment of experimental glioblastomas with anti-angiogenic drugs. We used Platelet Factor 4-DLR (PF4-DLR), a peptide derived by inserting DLR mutations on a PF4 47–70aa fragment from Platelet Factor 4 that potently inhibits angiogenesis [6].

Platelet Factor-4-DLR inhibits the binding of iodinated VEGF or FGF-2 to cell surface receptors at much lower concentrations than the unmodified peptide, and abrogates VEGF or FGF-2-induced endothelial cell proliferation [6]. This inhibitor has been widely used in human glioblastoma models, in which it significantly inhibits tumor angiogenesis and growth. However, depending on the dose used and the tumors stage at which it is administered, prolonged treatment with PF4-DLR alone or in combination leads to the development of drug resistance [7].

Receptors and intracellular kinases are involved in cancer progression, metastatic spread and the development of resistance to pharmacological treatments [8]. Integrin-linked kinase 1(ILK1) is a protein dependent kinase that regulates Akt activity [9] in a PI3K-dependent manner [10]. It is an important regulator of tumor proliferation, invasion and angiogenesis because it increases VEGF expression by stimulating HIF-1α via AKT phosphorylation on Ser^473^ [11]. It also promotes cell growth [12] and cell cycle progression [13], and inhibits apoptosis [14]. Recent studies have demonstrated that ILK1 is involved in glioblastoma progression [15] and radioresistance [16].

We found that ILK1 expression is down-regulated after ten days treatment and up-regulated after twenty days. Interestingly treatment with PF4-DLR and an anti-ILK1 short interfering RNA is associated with a decrease in tumor mass and a reduction in the number of tumor vessels.

Our findings have important therapeutic implications and suggest that combination strategies that simultaneously inhibit different mechanisms of tumor proliferation and angiogenesis may significantly increase therapeutic efficacy. We also analyzed the levels of ILK1 in patients with glioblastomas, astrocytomas and oligodendrogliomas, and found that high levels of ILK1 expression correlate with a poor prognosis. Our data suggest that ILK1 could represent a new specific pharmacological target to be inhibited alone or in combination with anti-angiogenic therapies in gliomas.

## Materials and Methods

### Ethics Statement

Animals were used in accordance with protocols approved by the Italian Minister for Scientific Research, protocol number 11/07, approved on February 28, 2007. Brain tumor tissue was obtained following written informed consent, approved by our Internal Ethical Committee (Neurosurgery, Ospedale Maggiore Policlinico, Milano).

### Cell cultures

The human glioma cell line U87-MG (American Type Culture Collection, Manassas, VA) was used in the animal experiments. The cells were cultured in αMEM (Life Technologies, Inc., Grand Island, NY) supplemented with 2 mM L-glutamine, 10% FBS, and 1000 units/ml gentamycin solution, maintained in T-25 tissue culture flasks in 5% CO2/95% air at 37°C in a humidified incubator. For the intracranial implantation experiments, U87-MG cells were dispersed with a 0.05% solution of trypsin/EDTA (Life Technologies), reaction was stopped with FBS. The cells were washed with PBS, and adjusted to a final concentration of 5×10^4^ cells/10 µl in PBS.

### Animal experiments

For the intracranial glioblastoma model, groups of ten 6-week-old nude mice (Charles Rivers Italia, Calco, Italy) were intracranically implanted with 50,000 human U87 glioma cells using an open window technique [17]. Twelve days after tumor cell injection (when an already well established and angiogenic tumor had developed), the animals were implanted subcutaneously with 2004 Alzet osmotic minipumps (ALZET, Cupertino, CA). The pump reservoir was filled with 0.5 mg of the peptide in PBS. The control groups received pumps containing PBS. The first group of animals was sacrificed 10 days after pump implantation, and the second group was sacrificed 20 days after pump implantation (see Table S1).

To investigate the effect of combined treatment with PF4-DLR and an anti-ILK1 small interference RNA, groups of 6-week-old nude mice were implanted intracranically with 50,000 human U87-MG glioblastoma cells (see above) and, twelve days after tumor cells injection, they were divided into three groups: one group was implanted with osmotic minipumps filled with 0.5 mg of PF4-DLR in PBS, and the animals were sacrificed 20 days after pump implantation; a second group was first implanted with pumps filled with 0.5 mg of PF4-DLR in PBS and, 10 days after the beginning of treatment with PF4-DLR ILK1-siRNA was administered intrathecally with a minipump, filled with 0.2 mg of siRNA in PBS,connected via a brain-infusion cannula stereotaxically placed and the combined treatment was continued for 10 days before the mice were sacrificed; finally the control group was implanted with pumps containing PBS and sacrificed 20 days after pump implantation (see Table S2).

At the time of sacrifice, fresh dissected brains were quickly frozen in liquid nitrogen and stored at −80°C. All of the animal experiments were repeated at least three times.

### Sample preparation for 2D-PAGE and gel analysis

All of the tissues were mechanically lysed at 4°C in CHAPS buffer [CHAPS 4%, 5 mM Tris, pH 8.8, 0.05% protease inhibitor cocktail (Sigma)] using a glass potter, and the samples were centrifuged at 2000 g for 15 minutes at 4°C in order to eliminate aggregates and debris and then stored at −20°C. The protein concentrations in each sample were measured by means of a DC Bio-Rad assay. A total of 900 µg of proteins from each sample was precipitated with cold acetone and resuspended in thiourea buffer (7 M urea, 2 M thiourea 2% CHAPS, 2% ASB-14, 5% glycerol, 40 mM DTT, 4 mM TCEP, 1% 3–10 IPG buffer, Amersham), and the samples were mixed overnight at 4°C in the dark and clarified by centrifugation at 16000 g for 15 minutes at 4°C. The supernatant was first separated by isoelectric focusing over a pH range of 3–10 using precast first-dimension drystrip 3–10 NL 18 cm (Amersham) following a multi-step protocol for 90,000 Vht (Protean IEF cell, Biorad). The first-dimension strip was equilibrated in 50 mM Tris, pH 8.8, 6 M urea, 30% glycerol, 2% SDS plus 16 mM DTT for 20 minutes, and then plus 25 mM iodioacetamide for 15 minutes, and loaded on a large format (22×22 cm) 9–16% acrylamide gel to separate the proteins by molecular weight. Second-dimension runs were performed using Biorad XL cells at 30 V 1 h, 300 V 4 h at a constant temperature of 18°C. Protein spots were revealed using home-made blue Coomassie staining, and the gel images were acquired by means of an Image scanner at 300 DPI resolution and analyzed using Image2D Master Platinum software (both from Amersham). At least seven gels per condition were included in the analysis.

Normalized spot volume values were studied using SPSS software version 13.0 for statistical analysis (SPSS Inc.). In brief, the data for each spot match set were analyzed using a box-plot test in order to eliminate outliers, a K-S test to check normal distribution, an F test to analyze the variance, and finally Student's t test to compare the mean values, which were considered significant when p<0.05.

### Trypsin digests of protein spots and MALDI-TOF-MS

Protein spots of interest were excised from the gels, reduced, alkylated, and digested overnight with bovine trypsin (Roche Diagnostic), as previously described [18], and a 1 µl aliquot of the supernatant underwent mass analysis using the dried droplet technique and a-cyano-4-hydroxycinnamic acid as matrix; the mass spectra were obtained using a MALDI–TOF Voyager- DE STR mass spectrometer (Applied Biosystems). Alternatively, gel fragments were further extracted and the resulting peptide mixture underwent a single desalting/concentration step before mass spectrometric analysis over mZipTipC18 (Millipore Corporation).

The spectra were internally calibrated using trypsin autolysis products, and processed using Data Explorer software. The proteins were unambiguously identified by searching against a comprehensive non-redundant protein database using the Pro-Found and

MASCOT programs [19]; one missed cleavage per peptide was allowed, and an initial mass tolerance of 50 ppm was used in all searches.

### Western blot analysis and antibodies

For Western blot analysis, 700 µg of proteins from each sample were precipitated twice in cold acetone 80% and 0.2 mM DTT for 30 minutes at 16,000 g and 4°C. The protein pellets were resupended in 150 µl of Laemmli's buffer, and 10 µl were loaded onto 6–12% SDS-PAGE gels. The proteins were transferred onto nitrocellulose membrane (Sigma) at 80 V for 120 minutes at 4°C. Primary antibodies were applied overnight in blocking buffer (20 mM Tris, pH 7.4, 150 mM NaCl, 0.1% Tween 20, and 3% dried non-fat milk). Antibodies against caspase 3 precursor, HSP90, PKC, ILK1, pAKT, AKT, VEGFR and EGFR (Cell Signaling), peEF2, eEF2 and eEF2 kinase (gifts from A. Nairm.), vinculin, tubulin and actin (Sigma), and the secondary antibodies (HRP-conjugated anti-mouse, anti-rabbit or anti-goat) (Amersham) were used in a ratio of 1∶2000. The signal was detected by an ECL detection system (PerkinElmer Life Sciences, Emeryville, CA), captured by means of a Versadoc 1000 digital camera (Biorad), and quantified using ImageQuant software (Bio-Rad).

### siRNA knockdown

The silencing ILK1 RNA stabilized oligonucleotides (siRNA: sense GCACCAAUUUCGUCGUGGAUU; antisense 5′-pUCCACGACGAAAUUGGUGCUU) were purchased from Dharmacon, and were used with a 21-bp non-silencing control sequence. For the *in vitro* study, U87-MG cells were transfected with lipofectamine 2000 (Invitrogen, Frederick, MD) following the manufacturer's instructions using 50 and 100 mM non-silencing or siRNA with 6 µl LipofectAMINE 2000 reagent for 400,000 cells. All of the experiments were performed 48 hours after transfection, and proliferation was assayed 96 hours after transfection. ON TARGET-plus siRNAs for ILK1 siRNA (sense GCACCAAUUUCGUCGUGGAUU; antisense 5′pUCCACGACGAAAUUGGUGCUU) or siNTC (Dharmacon) were used for the *in vivo* study. The siRNAs were administered at a concentration of 0.4 mg/kg/day in PBS.

### Proliferation assay

This assay was used to test the biological activity of human ILK1 siRNA on U87-MG cell proliferation. U87-MG cells were plated on a 96-well plate (20,000cells/well) and cultured in the presence of ILK1 siRNA (50 or 100 mM) and 10% serum for 24, 48 and 96 hours. The relative number of cells was calculated using the 3-(4,5-dimethylthiazol-2-yl)-2,5-diphenyltetrazolium bromide conversion assay (MTT test) (Promega, Madison, WI).

### Production of recombinant human PF4-DLR

The COOH-terminal peptide of human PF-4-DLR (PF-4 47–70DLR: NGRKICL-*DLR*APLYKKIIKKLLES) was synthesised on a peptide synthesizer using standard Merrifield solid-phase synthesis protocols and t-butoxycarbonyl chemistry, and analysed by means of reverse- phase HPLC chromatography. The peptides used for the biological assays were further purified by dialysis using Spectrapor 500 MW-cut-off membranes or by means of reverse-phase purification using CI8 Sep-Pak cartridges. The identity of the peptides was verified using MALDI-TOF-MS.

### Histology, immunofluorescence and immunochemistry

For the histology studies, 10 µm sections of brains removed from treated and untreated mice were fixed with methanol 100% and stained with hematoxylin and eosin (Bio-Optica). Images were acquired using a StemiDV4 microscope equipped with a AxioCam ICc 1 camera (Zeiss). Tumor volume were measured by the ellipsoid formula [(width^2^×length)/2]. Between-group differences in tumor growth were analyzed using the ANOVA with post hoc Tukey test, with p values of <0.05 being considered statistically significant.

For the immunofluorescence studies, 10 µm sections were fixed in 4% paraformaldehyde and 4% sucrose at room temperature for 10 minutes. The primary and secondary antibodies were applied in Gelatin Detergent Buffer (GDB) buffer (30 mM phosphate buffer, pH 7.4, containing 0.2% gelatin, 0.5% Triton X-100 and 0.8 M NaCl). The samples were stained using antibodies against rat anti-mouse CD31 (1∶50, B&D, Pharmigen, San Jose, CA). The secondary antibody used was anti-rat Cy3-conjugated (Jacson ImmunoResearch, West Grove, PA). All of the sections were counterstained with DAPI (Sigma). The fluorescence images were acquired using a confocal LSM510 Zeiss (gift from Monzino Foundation) with a Zeiss 20x objective. All of the measurements were made using Metamorph image analysis software (Universal Imaging Corporation), and were expressed as mean values ± standard error mean (SEM).

Immunohistochemical analysis was performed with the Vectastain ABC system (Vector Laboratories, Burlingame, CA). In brief, 10 µm sections were air dried and methanol fixed; endogenous peroxidase were blocked with 0.3% H_2_O_2_ in PBS for 30 minutes; primary antibodies against rat anti-mouse CD31 (1∶50, B&D, Pharmigen, San Jose, CA) or rabbit anti-human ILK1 antigens (1∶50 Cell Signaling) were applied in GDB buffer for 2 hours at room temperature. Secondary antibodies conjugated with biotin were applied in GDB buffer for 1 hour at room temperature. The slices were then incubated with ABC reagent for 30 minutes at room temperature and subsequently treated with DAB until brown staining appearance. Finally, the sections were stained with hematoxylin, dehydrated in ethanol and mounted with glass coverslips. The image were visualized with a Leica microscope equipped with 10x and 20x objectives and images were acquired using an AxioCam ICc 1 camera (Zeiss).

### Tissue samples

The tumor tissues came from patients with astrocytomas, oligodendrogliomas or glioblastomas, diagnosed on the basis of the latest WHO classification of gliomas, and were obtained at the time of surgery and stored in nitrogen vapor. Tissues were prepared for Western Blot analysis as described before.

For the immunohistochemistry tissues from patients with different types of tumors were analyzed by the Vectastain Elite ABC kit (Vector Laboratories, Burlingame, CA). All of the specimens were formalin fixed and paraffin embedded. Three 5 µm thick sections were cut from each block. The paraffin sections were deparaffined in xylene and antigen was retrieved using sodium citrate buffer (pH 6.0) in a microwave oven three times for five minutes each time. The sections were stained for immunohistochemical analysis with the primary anti-mouse ILK1 antibody (see above).

### Statistical analysis

Data were expressed as means with standard errors of the mean (SEM). The significance of differences was tested by unpaired Student t test (two means) or ANOVA with post hoc Tuckey test (more than two means). The SPSS statistical package version 13.0 (SPSS Inc.) was used for the analyses.

## Results

### Proteomic analysis of experimental glioblastoma treated with PF4-DLR

In order to identify the changes in protein expression induced by PF4-DLR treatment, nude mice bearing U87-MG glioblastoma xenografts were treated using osmotic minipumps that released 0.5 mg/kg/day of peptide. Pumps were implanted 12 days after tumor cells implantation. Animals were sacrificed for the proteomic analysis 10 and 20 days later.

Two-dimensional SDS polyacrylamide gel electrophoresis (2D-SDS-PAGE) was used to compare protein expression patterns in treated and untreated tumors of the same age.

**Figure 1:**
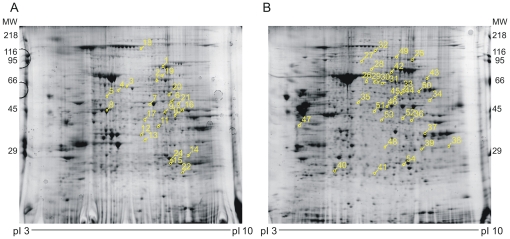
Two-dimensional PAGE gels analysis of PF4-DLR treated glioblastomas. In order to identify protein expression changes in mice treated with PF4-DLR, samples of treated and untreated glioblastomas were analyzed by means of two-dimensional PAGE gels. The tissues were prepared as described in Materials and Methods: 900 µg of protein underwent two-dimensional PAGE using a first dimension pH NL gradient of 3-10% and a second dimension gradient of 9–16% acrylamide. The gels were stained with colloidal Coomassie blue. A) example of gel obtained from glioblastomas treated with PF4-DLR for 10 days; B) example of gel obtained from glioblastomas treated with PF4-DLR for 20 days. At least seven gels per condition were used to analyze protein changes. The analyzed spots and related identification numbers are shown in yellow.

At least seven independent gels were used for matching in each group, and a total of more than 1800 protein spots were analyzed (Figures 1A–B and Figure S1A–B show typical 2D gels). The gels included in the analysis were chosen on the basis of comparable total spot numbers, and percentages and distribution parameters as detected by the Image2D Master Platinum software (Amersham).

In tumors treated with PF4-DLR for 10 days, 24 proteins were differentially expressed (p<0.05) in response to the treatment. Of these, 14 were up-regulated and 10 down-regulated (Figure 1A, Table 1 and Figure S2). A set of proteins that correlate with the inhibition of tumor growth in the response to therapy included transforming growth factor-β induced (TGFBI), annexin A1 and protein disulfide-isomerase, which were increased. Another set of proteins involved in cell proliferation was also increased after 10 days of treatment. These include serotransferrin precursor, bifunctional purine biosynthesis protein, elongation factor 1, vinculin and septin 5 (see Table 1 and Figure S2).

**Table 1:** DLR 10 days.

Swiss prot TrEMBL Acc No	Protein name	Spot No	Mw	pI	DLR/Ctr	Peptides matched	Sequence coverage
Q15063	Serotransferrin precursor	1	76.7	6.94	1.6 (p<0.05)	35/24	36.4%
P31939	Bifunctional purine biosynthesis protein	2	64.6	6.27	1.9 (p<0.05)	4/4	8.4%
O08553	Dihydropyrimidinase-related protein 2	3	61.1	5.95	0.4 (p<0.001)	20/12	28.8%
P30101	Protein disulfide-isomerase A3 precursor	4	56.7	5.98	2.0 (p<0.05	21/15	33.7%
Q9D1A2	Cytosolic nonspecific dipeptidase	5	52.7	5.43	0.6 (p<0.05)	8/8	17.0%
P06733	Alpha-enolase	6	47.0	7.01	1.9 (p<0.05)	16/7	22.6%
P26641	Elongation factor 1-gamma	7	50.0	6.25	2.3 (p<0.05)	15/10	24.5%
Q04447	Creatine kinase B-type	8	42.7	5.40	0.4 (p<0.005)	30/10	34.9%
P15105	Glutamine synthetase	9	42.0	6.47	0.6 (p<0.005)	11/7	25.3%
P05201	Aspartate aminotransferase	10	46.1	6.68	0.6 (p<0.05)	17/11	30.6%
P04083	Annexin A1	11	38.6	6.57	2.0 (p<0.005)	24/17	46.4%
P14152	Malate dehydrogenase	12	36.3	6.16	0.5 (p<0.005)	22/7	29.7%
P53810	Phosphatidylinositol transfer protein alpha	13	31.7	5.97	0.7 (p<0.05)	12/6	28.9%
Q9R1P0	Proteasome subunit alpha type 4	14	29.4	7.58	1.9 (p<0.05)	3/3	10.3%
P60174	Triosephosphate isomerase	15	26.5	6.45	1.7 (p<0.005)	23/10	41.1
P00558	Phosphoglycerate kinase	16	44.5	8.30	0.6 (p<0.005)	7/7	25.0%
Q9Z2Q6	Septin-5	17	42.7	6.21	0.7 (p<0.05)	4/4	13.8
P18206	Vinculin	18	123.6	5.50	1.6 (p<0.05)	20/20	22.7
Q15582	Transforming growth factor-beta-induced	19	74.6	7.62	2.2 (p<0.05)	9/8	13.8%
Q16658	Fascin	20	54.4	6.84	2.4 (p<0.005)	9/9	20.1%
Q9CZU6	Citrate synthase	21	51.7	8.72	2.4 (p<0.05)	5/5	12.7%
P19157	Glutathione S-transferase P 1	22	23.5	7.69	0.4 (p<0.05)	6/5	32.1%
P62334	26S protease regulatory subunit S10B	23	44.1	7.09	1.8 (p<0.001)	7/7	20.8%
P60174	Proteasome subunit alpha type 6	24	26.5	6.45	1.7 (p<0.005)	19/10	43.1%

List of differentially expressed proteins identified by MALDI-TOF after two- dimensional PAGE analysis of glioblastomas treated with PF4-DLR for ten days or left untreated. The experimental MW and pI are indicated for each protein.

Tumors treated with PF4-DLR for 20 days showed 30 differentially expressed proteins (p<0.05). Twenty four were up-regulated and 6 down-regulated in treated tumors (Figure 1B, Table 2 and Figure S3). Among these proteins, integrin-linked kinase 1 (ILK1), stress-inducible protein 1 (STI1), eukaryotic elongation factor 2 (eEF2), vinculin, fascin, pyruvate kinase M2, transketolase, moesin, and the plasminogen precursor were found (see Table 2 and Figure S3).

**Table 2:** DLR 20 days.

Swiss prot TrEMBL Acc No	Protein name	Spot No	Mw	pI	DLR/Ctr	Peptides matched	Sequencecoverage
P13639	Elongation factor 2	25	95.3	6.41	1.8 (p<0.05)	72/28	33%
P02545	Lamin A/C	26	74.1	6.57	1.4 (p<0.01)	13/13	20%
P06396	Gelsolin precursor	27	85.7	5.90	1.7 (p<0.001)	3/2	3%
P41250	Glycyl-tRNA synthetase	28	83.1	6.61	1.5 (p<0.05)	13/9	11%
P02545	Lamin A/C	29	74.1	6.57	1.6 (p<0.001)	17/14	22%
P02545	Lamin A/C	30	74.1	6.57	1.4 (p<0.001)	29/21	31%
P02545	Lamin A/C	31	74.1	6.57	1.9 (p<0.001)	22/17	30%
P18206	Vinculin	32	123.8	5.50	2.5 (p<0.001)	55/30	31%
P14618	Pyruvate kinase	33	58.0	7.96	1.5 (p<0.01)	16/11	23%
Q13418	Integrin-linked protein kinase	34	51.4	8.30	2.0 (p<0.05)	2/2	5%
P61158	Actin-like protein 3	35	47.4	5.61	1.4 (p<0.05)	16/12	38%
P17174	Aspartate aminotransferase	36	46.2	5.53	1.7 (p<0.05)	29/12	29%
P04406	GAPDH	37	36.0	8.57	0.6 (p<0.01)	13/5	23%
P21796	hVDAC-1	38	30.1	8.62	0.6 (p<0.05)	8/7	30%
P09651	hnRNP A1	39	38.8	9.26	2.3 (p<0.005)	5/4	13%
Q00623	Apolipoprotein A-I precursor	40	30.6	5.64	1.6 (p<0.05)	26/9	33%
P61106	Ras-related protein Rab-14	41	23.4	5.85	0.6 (p<0.05)	11/10	44%
P26038	Moesin	42	67.8	6.08	2.7 (p<0.01)	62/25	30%
P29401	Transketolase	43	67.9	7.58	1.8 (p<0.05)	56/16	27%
P12268	Inosine-5′-monophosphate dehydrogenase 2	44	55.8	6.44	2.0 (p<0.005)	21/10	23%
Q16658	Fascin	45	54.5	6.84	1.8 (p<0.05)	5/5	8%
P26641	Elongation Factor 1-gamma	46	50.1	6.25	1.9 (p<0.05)	27/11	17%
P09493	Tropomyosin alpha-1 chain	47	32.7	4.69	1.5 (p<0.05)	13/11	33%
P42574	Caspase-3 precursor	48	31.6	6.09	0.5 (p<0.05)	4/2	11%
P20918	Plasminogen precursor NB	49	90.8	6.21	1.4 (p<0.05)	7/6	7%
P31948	Stress-induced.phosphoprotein 1	50	62.6	6.40	1.8 (p<0.05)	24/13	27%
P23526	Adenosylhomocysteinase	51	47.7	5.92	1.6 (p<0.05)	35/15	33%
P49411	Elongation Factor Tu	52	49.5	7.26	2.0 (p<0.001)	2/2	5%
P61160	Actin-like protein 2	53	44.8	6.29	0.7 (p<0.05)	7/4	12%
P13634	Carbonic anhydrase	54	28.3	6.44	0.5 (p<0.01)	4/4	24%

List of differentially expressed proteins identified by MALDI-TOF after two dimensional PAGE analysis of glioblastomas treated with PF4-DLR for 20 days or left untreated. The experimental MW and pI are indicated for each protein.

### Confirmation of protein expression changes by Western blotting

**Figure 2:**
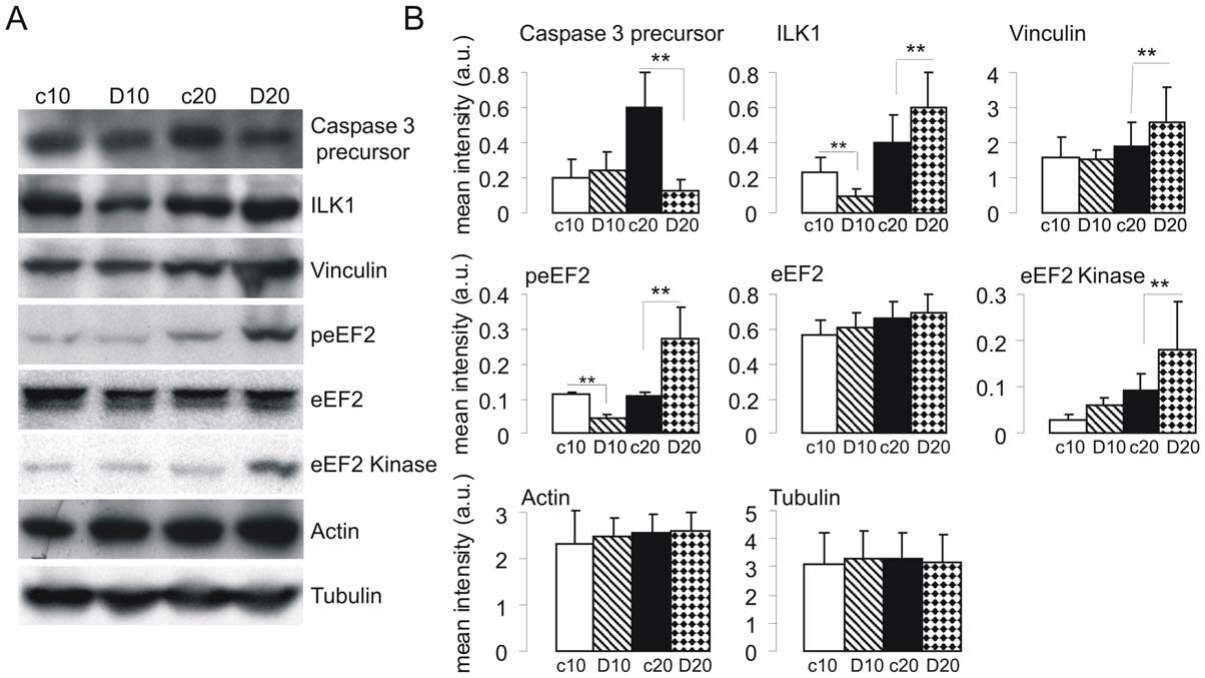
Immunoblotting validation of a set of proteins identified as being differentially expressed in glioblastomas treated with PF4-DLR. Tissue lysates of glioblastomas treated with PF4-DLR for 10 or 20 days (D10 and D20) or untreated (c10 and c20) were prepared as described in Materials and Methods, and the proteins were resolved on 6–12% PAGE gels. A) The proteins were blotted onto a nitrocellulose membrane and detected with antisera raised against the individual proteins indicated on the right side of each panel. B) Histograms of the mean band intensity (± SEM) of each protein. *p<0.05, t test.

As part of validation of the proteomic analysis, the expression of some of the identified proteins was tested by immunoblotting using protein extracts from glioblastomas treated with PF4-DLR for 10 and 20 days. As expected, caspase 3 was decreased after 20 days of PF4-DLR treatment when compared to untreated tumors (Figure 2A and 2B), in agreement with the proteomic analysis. Immunoblotting not only confirmed the up-regulation of ILK1 in tumors treated with PF4-DLR for 20 days, but also revealed a significant decrease in ILK1 levels after 10 days of treatment in comparison to untreated tumors (Figure 2A and 2B).

2D gels showed three spots corresponding presumably to eEF2 protein with the same molecular weight but with different pI values. The treated tumors showed a significant increase in the more acidic spots. This may be due to a post-translational modification such as protein phosphorylation. We therefore analyzed the phosphorylation level of eEF2 using anti-phospho-eEF2 and total eEF2 antibodies. As shown in Figure 2A and 2B, eEF2 phosphorylation was significantly reduced after 10 days of PF4-DLR treatment, but also significantly increased after 20 days. In addition, we investigated the expression of eEF2 kinase and found a significant increase after 20 days.

Finally, immunoblotting confirmed the increase in expression of vinculin in mice treated for 20 days (Figure 2A and 2B).

### PF4-DLR specifically induces ILK1 pathway activation

**Figure 3:**
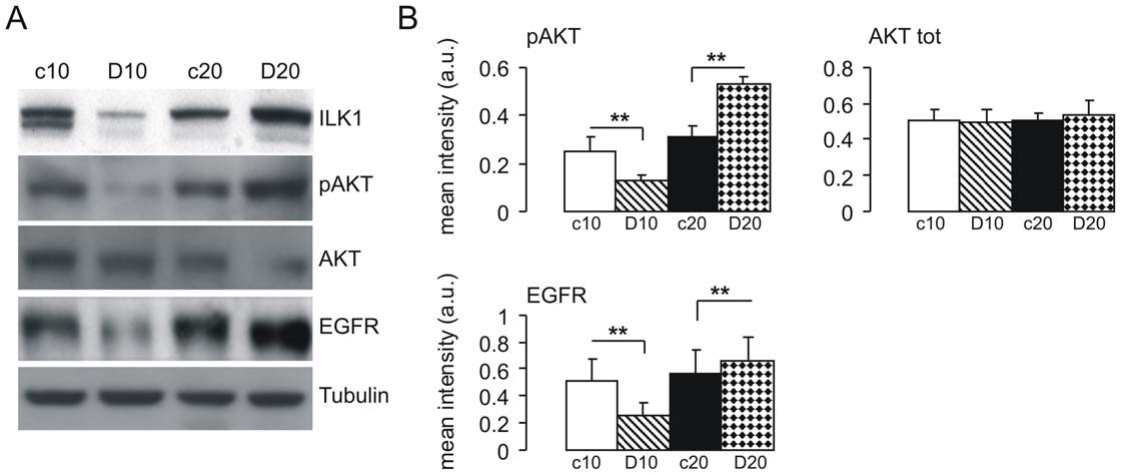
Immunoblotting analysis of ILK1, pAKT and EGFR in PF4-DLR treated glioblastomas. Tissue lysates of the treated (D10 and D20) and untreated (c10 and c20) tumors were prepared as described in Materials and Methods, and the proteins were resolved on 10–12% PAGE gels. A) The proteins were blotted onto a nitrocellulose membrane and detected with antibodies raised against the individual proteins indicated on the right side of each panel. B) Histograms of the mean band intensity (± SEM) of each protein. *p<0.05, t test.

Among the identified proteins, we decided to concentrate on ILK1. The kinase activity of ILK1 is stimulated by growth factors and chemokines in a PI3 kinase-dependent manner [10]. ILK1 can also mediate the phosphorylation of a variety of intracellular substrates, particularly protein kinase B/Akt on serine-473 [20]. We therefore, tested the levels of AKT phosphorylation in tumors treated with PF4-DLR. As expected, AKT phosphorylation was decreased after 10 days of treatment and increased after 20 days (Figures 3A and 3B).

It has been shown that growth factors receptor expression correlates with ILK1 expression [21]. We therefore analyzed the levels of EGFR by immunoblotting: similar to ILK1 and pAKT expression of EGFR was reduced after 10 days of PF4-DLR treatment and increased after 20 days (Figures 3A and 3B). On the contrary, VEGFR2 was down-regulated by PF4-DLR after both 10 and 20 days of treatment (Figure 3A and 3B).

This may indicate that PF4-DLR, after 10 days of treatment, is decreasing ILK1 levels by down-regulating EGFR. On the contrary, longer treatment (20 days), led to an increase in ILK1 levels, and of down-stream pathways.

### ILK1 knockdown in glioblastoma cells increases PF4-DLR activity

**Figure 4:**
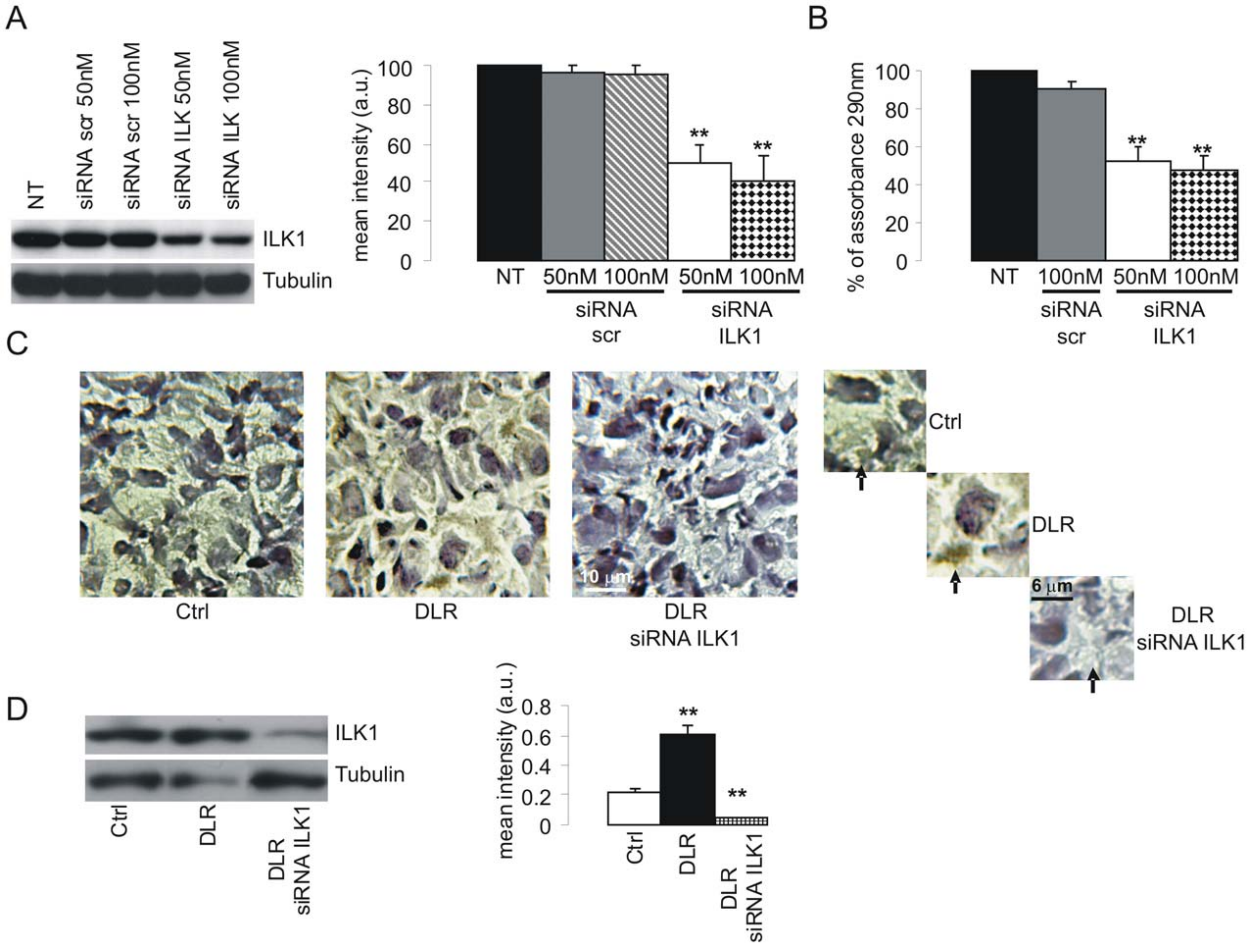
Characterization of specific siRNA for ILK1. A) U87 cells were transiently transfected with ILK1-specific siRNA using Lipofectamine 2000 or nontransfected (NT) as described in Materials and Methods, and harvested 48 h later; untreated cells or a non-silencing scrambled siRNA (Scr) were included as controls. ILK1 expression was specifically reduced in the cells transfected with ILK1 siRNA. Histogram shows of the mean band intensity (± SEM) of each protein. **p<0.01, one-way ANOVA with post hoc Tuckey test.B). Effect of the administration of ILK1 siRNA *in vitro* using the MTT test of cell proliferation. ILK1 siRNA 50 nM and 100 nM respectively led to the 48% and 53% inhibition of cell proliferation (columns 3 and 4); 100 nM of scrambled siRNA (Scr 100 nM) did not reduce cell proliferation (compare columns 1 and 2). NT: not transfected. Histogram shows mean % of assorbance at 290 nm. **p<0.01, one-wayANOVA with post hoc Tuckey test. C and D) Effect of the administration of ILK1 siRNA *in vivo*; representative immunohistochemistry (C) (Scale bar  = 10 µm) and Western Blot (D) analysis of ILK1 expression in tumors from untreated mice (ctrl), and mice treated with PF4-DLR alone (DLR) or PF4-DLR plus ILK1 siRNA (DLR siRNA ILK1). ILK1 levels were significantly reduced in the mice treated with ILK1 siRNA. Histogram in D shows of the mean band intensity (± SEM) of each protein. **p<0.01, one-way ANOVA with post hoc Tuckey test.

We next performed knocked down experiments using specific ILK1 siRNA and analyzed its consequences on glioma cell proliferation and tumor development in vitro and in vivo. The ability of ILK1-siRNA to reduce the expression of ILK1 protein was first tested in U87-MG cells in vitro. Both 50 and 100 nM ILK1 siRNA reduced ILK1 protein expression (NT: 100%; scrambled 50 nM: 96.6%; scrambled 100 nM: 94.9%; siRNA ILK1 50 nM: 50.0%; siRNA ILK1 100 nM: 41.3%) (Figure 4A). We next measured U87 –MG proliferation. As expected ILK1 siRNA reduces cell proliferation (NT: 100%; scrambled 100 nM: 90.0%; siRNA ILK1 50 nM: 52.0; siRNA ILK1 100 nM: 47.2) to about 50% when incubated for 48 hours in comparison with cells transfected with scrambled control siRNA (Figure 4B).

We then tested whether the inhibitory activity of PF4-DLR can be increased in vivo by combined treatment with PF4-DLR and ILK1 siRNA in the experimental glioma model in mice. A first osmotic mini-pump releasing PF4-DLR (0.5 mg/kg/day) was implanted 12 days after tumor injection, and followed, after additional ten days, by the implantation of a second minipump, intrathecally connected, releasing ILK1 siRNA (0.4 mg/day). Animals were sacrificed 10 days later. As control, some mice were left untreated and others were treated with PF4-DLR alone for 20 days (see Table S2). As shown in Figure 4C and 4D, the expression of ILK1 was greatly reduced in tumors when mice were treated with ILK1 siRNA.

**Figure 5:**
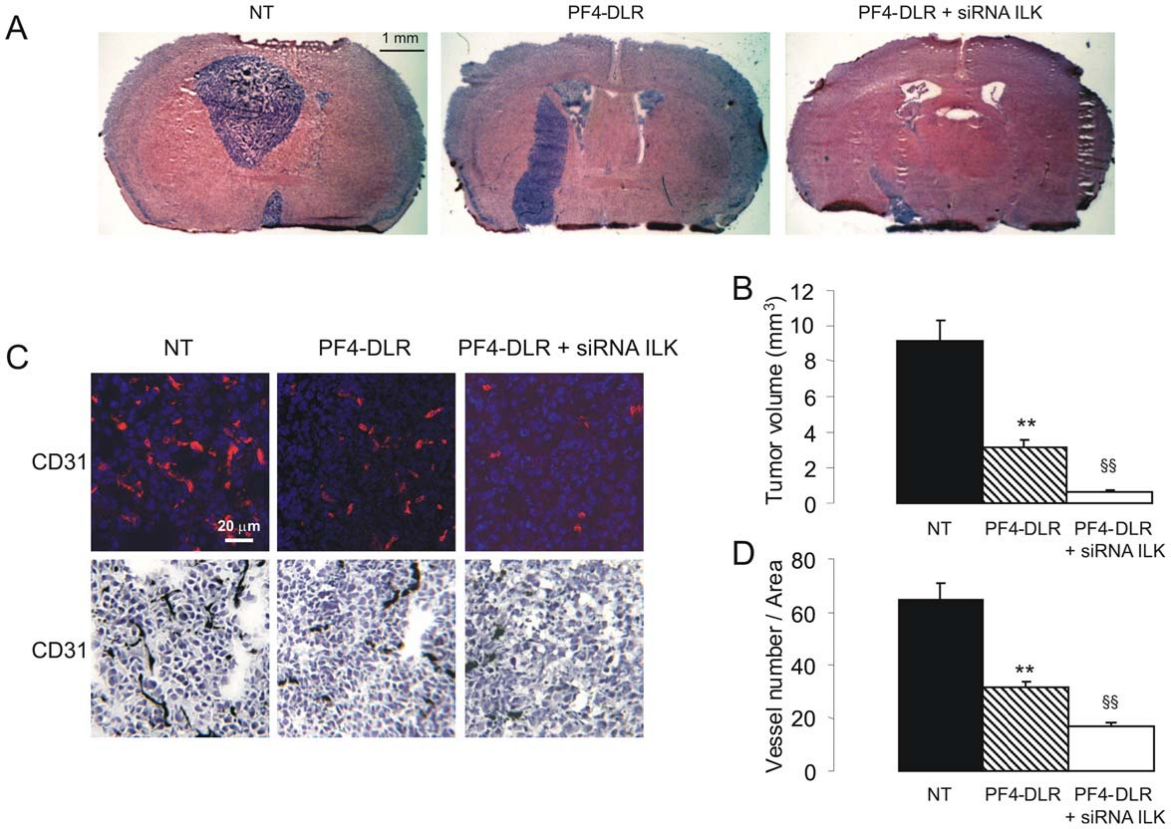
Effect of PF4-DLR + siRNA ILK1 treatment on glioblastomas development in vivo. A) Representative hematoxylin-eosin stained histological sections of brains from mice xenografted with U87-MG and treated or not (NT) with PF4-DLR or PF4-DLR plus ILK1 siRNA (PF4-DLR + siRNA ILK1). Scale bar  = 1 mm. B) Tumor volumes were quantified from six different brains per group, and the results expressed as mean values ± SEM. Significant differences between groups are indicated (one-way ANOVA followed by Tukey's *post hoc* test, **p< 0.05 PF4-DLR versus NT; §§p< 0.05 PF4-DLR + siRNA ILK1 versus PF4-DLR). C) Representative image of vessel staining (blue: DAPI, red: anti CD31) and immunohistochemestry of CD31. Scale bar 20 µm. D) Vessels were counted in the tumor area and were found significantly reduced in animals treated with PF4-DLR or PF4-DLR plus ILK1 siRNA (PF4-DLR + siRNA ILK1). Significant differences between groups are indicated (one-way ANOVA followed by Tukey's *post hoc* test **p<0.05 PF4-DLR versus NT; §§p<0.05 PF4-DLR + siRNA ILK1 versus PF4-DLR).

Untreated mice developed a large and compact tumor masses occupying about half of the brain with a smooth and regular margin, whereas treatment with PF4-DLR alone reduced tumor volume to ∼30% of untreated control. Combined treatment with PF4-DLR and ILK1-siRNA further reduced the size to ∼6% of untreated control (NT: 9.1±1.1; DLR: 3.2±0.4: DLR+ILK1 siRNA: 0.6±0.07 mm^3^) (Figure 5A and 5B). The effect of PF4-DLR and ILK1 siRNA on tumor angiogenesis was also evaluated by staining with anti-CD31 antibody. PF4-DLR alone significantly reduced the number of vessels, but the reduction was even greater after the combined treatment (NT: 64.8±6.5; DLR: 31.8±2.1; DLR+ILK1 siRNA: 17.1±1.5) (Figure 5C and D).

### ILK1 expression in human gliomas

**Figure 6:**
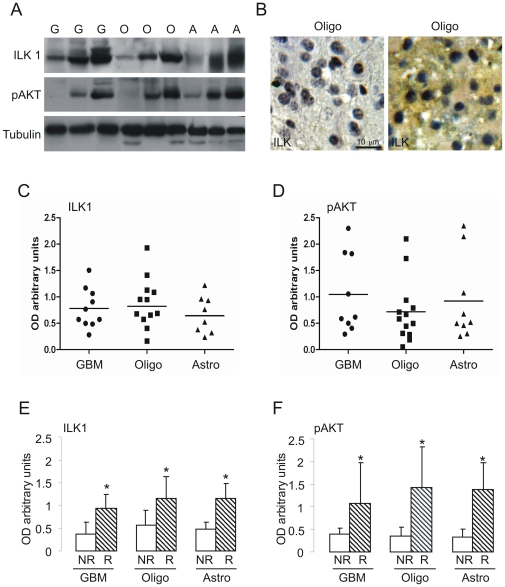
Immunoblotting analysis of ILK1 and pAKT levels in biopsies from patients. The expression level of ILK1 and pAKT were analyzed in biopsies from patients with glioblastomas (G), oligodendrogliomas (O) or astrocytomas (A). The tissue lysates were prepared as described in Materials and Methods, and the proteins separated in 10% gels. A) Proteins blotted onto a nitrocellulose membrane and detected using antibodies raised against the individual proteins indicated on the left side of each panel. B) Representative immunohistochemistry of ILK1 levels in different biopsies. C and D) The graphs show histological ILK1 and pAKT levels in different patients with the same type of tumor. E and F) Patients divided on the basis of the time of recurrence (NR: long-term recurrence, more than one year after treatment; R: short-term recurrence, less than four months after treatment). The histograms indicate the mean band intensity (± SEM) for each protein. *p<0.05, t test. The expression of ILK1 and pAKT was significantly upregulated in the subset of patients with a poor prognosis, regardless of the type of glioma.

We measured ILK1 expression in human glioblastomas (GBM), astrocytomas and oligodendrogliomas by immunoblotting. Ten GBM, 12 oligodendrogliomas and 8 astrocytomas were analyzed. Expression of ILK1 and its major substrate, pAKT, varied as shown in Figure 6A and 6C. Tumors expressed low, medium or high levels of ILK1 and pAKT, and this was confirmed by immunohistochemistry (Figure 6B shows two different oligodendrogliomas expressing low and high levels of ILK1).

We also correlated ILK1 expression levels of tumors to the time of recurrence (NR: long-term recurrence, more than one year after initial treatment, R: short-term recurrence, less than four months after initial treatment). ILK1 was significantly more expressed in the subset of patients with early recurrence in all three types of glioma (Figures 6E and 6F; astrocytomas: NR 0.47±0.12 R 2.51±0.9; oligodendrogliomas NR 0.6±0.13 R 1.2±0.2; GBMs NR 0.4±0.07 R 0.9±0.15). This suggests that gliomas expressing high ILK1 levels are more aggressive.

## Discussion

Previous experiments have shown that PF4-DLR reduces angiogenesis and inhibits tumor growth in a dose-dependent manner in the U87-MG model. We identified 24 proteins that were differentially expressed in the tumors treated with PF4-DLR for 10 days (14 up-regulated and 10 down-regulated), some of which are known to be involved in tumor development, and may constitute potential prognostic markers or therapeutic targets.

Annexin A1 (ANXA1), which plays an important role in regulating cell growth and apoptosis, was increased in treated tumors. Reduced ANXA1 expression has been observed in many different human malignancies, and the overexpression of ANXA1 in tumor cells leads to growth suppression and/or apoptosis [22].

Tumor growth and differentiation closely correlate with protein synthesis and degradation [23]–[24].We detected a significant increase of proteasome subunit alpha type 6, proteasome subunit alpha type 4 and 26S protease regulatory subunit S10B after PF4-DLR treatment. This indicates that anti-angiogenic treatment may induce molecular and functional changes in the intra-cellular proteolytic machinery.

Human glutathione *S*-transferase P1 (GSTP1) is highly expressed in many human cancers including gliomas [25]. High GSTP1 expression is associated with drug resistance, a more aggressive clinical course, and poor patient survival. The GSTP1 plays a major role in metabolism and xenobiotic detoxification, in which it catalyses the *S*-conjugation of a wide variety of endogenous and exogenous compounds [26]. There is also evidence indicating that it plays a role in many other important cellular processes, including stress, growth factor-induced signalling, cell proliferation, immune response, differentiation, cell transformation and apoptosis [27].

The GSTP1 is phosphorylated by Ser/Thr protein kinases, PKA and PKC, and this enhances its metabolic function [28]. We found that the more acidic form of GSTP1 was reduced in glioblastomas treated with PF4-DLR for 10 days. Interestingly, Western blot analysis revealed a decrease in PKC levels which suggests that PF4-DLR treatment regulates GSTP1 function by reducing its phosphorylation.

After 20 days of treatment, glioblastomas are still responsive to PF4-DLR. However, if treatment is prolonged, glioblastomas start to activate new pathways which may induce drug resistance. We identified 30 proteins that were differentially expressed (p<0.05) after 20 days of PF4-DLR treatment of which 24 were up-regulated and six down-regulated (Figure 1B and Table 2). As most of these proteins seem to be functionally involved in positively regulating tumor growth, glioblastomas may develop resistance to PF4-DLR by regulating this specific set of proteins.

There was a significant increase of the most acidic of the three spots corresponding to elongation factor 2 (eEF2). This is compatible with post-translational modifications of eEF2 through phosphorylation after PF4-DLR treatment, as previously demonstrated for eEF2. There is evidence that CaM-dependent phosphorylation of eEF2 is associated with cell proliferation in rat glia and gliomas [29]. We found that eEF2K expression is only increased by PF4-DLR treatment after 20 days. We also found an up-regulation of vinculin and fascin, which are key elements of cell motility. Increased expression of these proteins promotes cell migration and metastases [30].

The significance of ILK1 expression after PF4-DLR treatment was investigated in greater detail. ILK1 was down-regulated in glioblastomas treated with PF4-DLR for 10 days, but up-regulated after 20 days. This suggested that ILK1 expression correlates with treatment response, at least in our experimental model. ILK1 is a protein involved in intracellular signal transduction of integrins and growth factor receptors. In some tumors, increased ILK1 levels are required for cell growth/survival, cell cycle progression, invasion and migration, and tumor angiogenesis [21], [31], [32]. ILK1 overexpression in tumor cells induces the acquisition of an invasive phenotype and cell transformation. The role and regulation of ILK1 in gliomas has only been partially studied. A link with tumor cell invasion has been proposed [33], [34]. Furthermore, it can interfere with the cellular response to anti-cancer drugs such as resistance to gemcitabine in adenocarcinoma, an effect that is mediated by protein kinase B/Akt [35]. In a recent study, Edwards *et al*. found that inhibiting ILK1 with small molecule inhibitors causes reduced hypoxia, decreases tumor vascular mass and decreases functional vasculature in a mouse model of glioblastoma [36]. Interestingly ILK1 increases the expression of VEGF which imply that ILK1 might represent a key molecule for a positive loop inducing angiogenesis and tumor grown.

We found an association between ILK1 levels and AKT phosphorylation at Ser^473^, which indicates that PF4-DLR treatment for 10 days reduces glioblastomas growth by inhibiting the AKT pathway. As epidermal growth factor (EGF) is a key player in neoplastic progression [37] and AKT is a key effector of EGFR signalling, we analyzed EGFR levels in glioblastoma treated with PF4-DLR. Similar as observed with ILK1, 10 days of treatment led to EGFR down-regulation, but 20 days of treatment led to an increase in expression. The pattern of ILK1 expression after PF4-DLR treatment therefore correlates with AKT phosphorylation and EGFR expression. Interestingly it has been shown that ILK1 expression is induced by EGF [21], thus our data might suggest that PF4-DLR is able to increase the expression of EGFR which in turn can increase the response to EGF and the expression of ILK1.

The approach to target ILK1 is also supported by Edwards et al. They [36] demonstrated that inhibition of ILK1 alone is able to delay but not to stop tumor grown. We therefore decided to inhibit ILK1 using siRNA in addition to PF4-DLR administration in order to investigate whether this will further improve therapeutic efficacy in vivo over PF4-DLR alone.

At this point we need to clarify if after 10 days of treatment with PF4-DLR alone it is required to continue the anti-angiogenic therapy in association with ILK1 knock down or it is sufficient to continue only knocking down ILK1 to block or eradicate the tumor. In any case we think that the most important point is design effective combination treatment; this because the current opinion is that to eradicate tumor a single agent therapy is not enough.

We believe that the synergism of angiogenesis inhibitors with ILK1 inhibitors (such as PF4-DLR and ILK1 siRNA) should be tested using small molecular inhibitors blocking ILK1 function and not siRNA. Synthetic siRNA are extremely expensive and not likely to be used for systemic administration in the clinic.

It has been found that the overexpression of kinase-inactive ILK increases the sensitivity of EGFR-resistant hepatoma cell lines to the EGFR inhibitors erlotinib, gefitinib and cetuximab *in vitro* and *in vivo* [38]. These data demonstrate that inhibiting ILK activity may represent a novel mechanism for overcoming resistance to EGFR inhibitors.

ILK1 expression often correlates with the malignancy and poor survival in a number of tumors [39] including gliomas [34]. In our study, ILK1 expression seems to be associated with early recurrence in 3 glioma types. This finding suggests that ILK1 levels are related to tumor aggressiveness and its tendency to progress.

In conclusion, we found that ILK1 is involved in tumor response to anti-angiogenic therapy with PF4-DLR in a xenografted model of glioblastoma. Furthermore, its expression seems to be associated with the aggressiveness of the disease. Combined therapy associating anti-angiogenesis with inhibition of ILK1 may significantly enhance efficacy. This could represent a new venue for the development therapies for malignant glioma.

## Supporting Information

Figure S1 Two-dimensional PAGE gels analysis of PF4-DLR treated glioblastomas. In order to identify protein expression changes in mice treated with PF4-DLR, samples of treated and untreated glioblastomas were analyzed by means of two-dimensional PAGE gels. A) example of gel obtained from untreated glioblastomas which has been compared with PF4-DLR treated tumor for 10 days, same age; B) example of gel obtained from untreated glioblastomas which has been compared with PF4-DLR treated tumor for 20 days, same age.
(8.01 MB TIF)

Figure S2 List of differentially expressed proteins identified by MALDI-TOF after two- dimensional PAGE analysis by comparing glioblastomas treated with PF4-DLR for 10 days with corresponding untreated tumors. The graph indicates the ratio PF4-DLR treated/untreated tumors (PF4-DLR/Crtl) normalized spot volume values of the identified proteins.
(3.13 MB TIF)

Figure S3 List of differentially expressed proteins identified by MALDI-TOF after two- dimensional PAGE analysis by comparing glioblastomas treated with PF4-DLR for 20 days with corresponding untreated tumors. The graph indicates the ratio PF4-DLR treated/untreated tumors (PF4-DLR/Crtl) normalized spot volume values of the identified proteins.
(2.68 MB TIF)

Table S1 Proteomic analysis scheme.
(0.04 MB DOC)

Table S2 ILK1 knockdown experiment scheme.
(0.03 MB DOC)
