# Supplementary figures and images for: Anti-Angiogenic Therapy Induces Integrin-Linked Kinase 1 Up-Regulation in a Mouse Model of Glioblastoma
Source: PLoS One. 2010 Oct 29;5(10):e13710. doi: 10.1371/journal.pone.0013710 (PMC2966411; doi:10.1371/journal.pone.0013710)

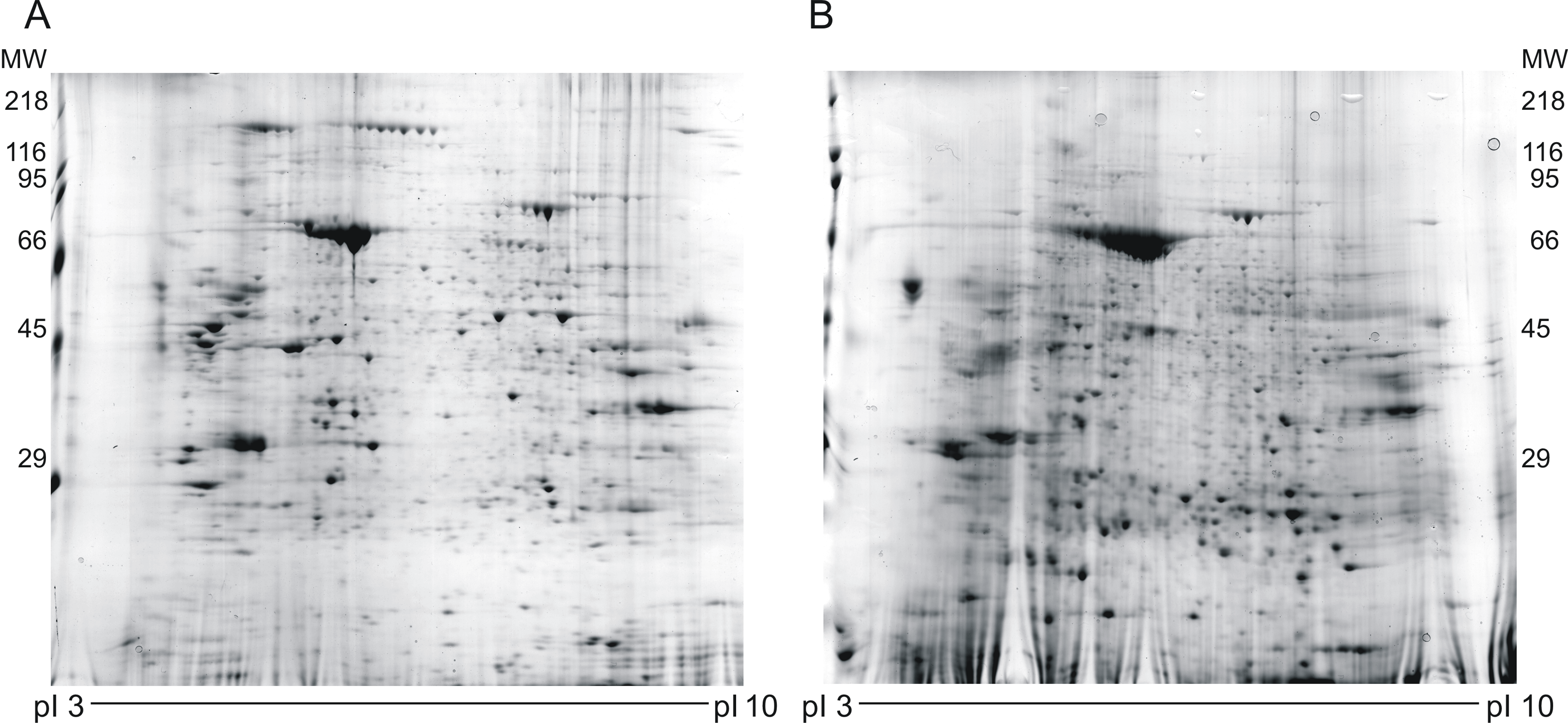

Supplement: Figure S1 — Two-dimensional PAGE gels analysis of PF4-DLR treated glioblastomas. In order to identify protein expression changes in mice treated with PF4-DLR, samples of treated and untreated glioblastomas were analyzed by means of two-dimensional PAGE gels. A) example of gel obtained from untreated glioblastomas which has been compared with PF4-DLR treated tumor for 10 days, same age; B) example of gel obtained from untreated glioblastomas which has been compared with PF4-DLR treated tumor for 20 days, same age. (8.01 MB TIF) [file pone.0013710.s001.tif]

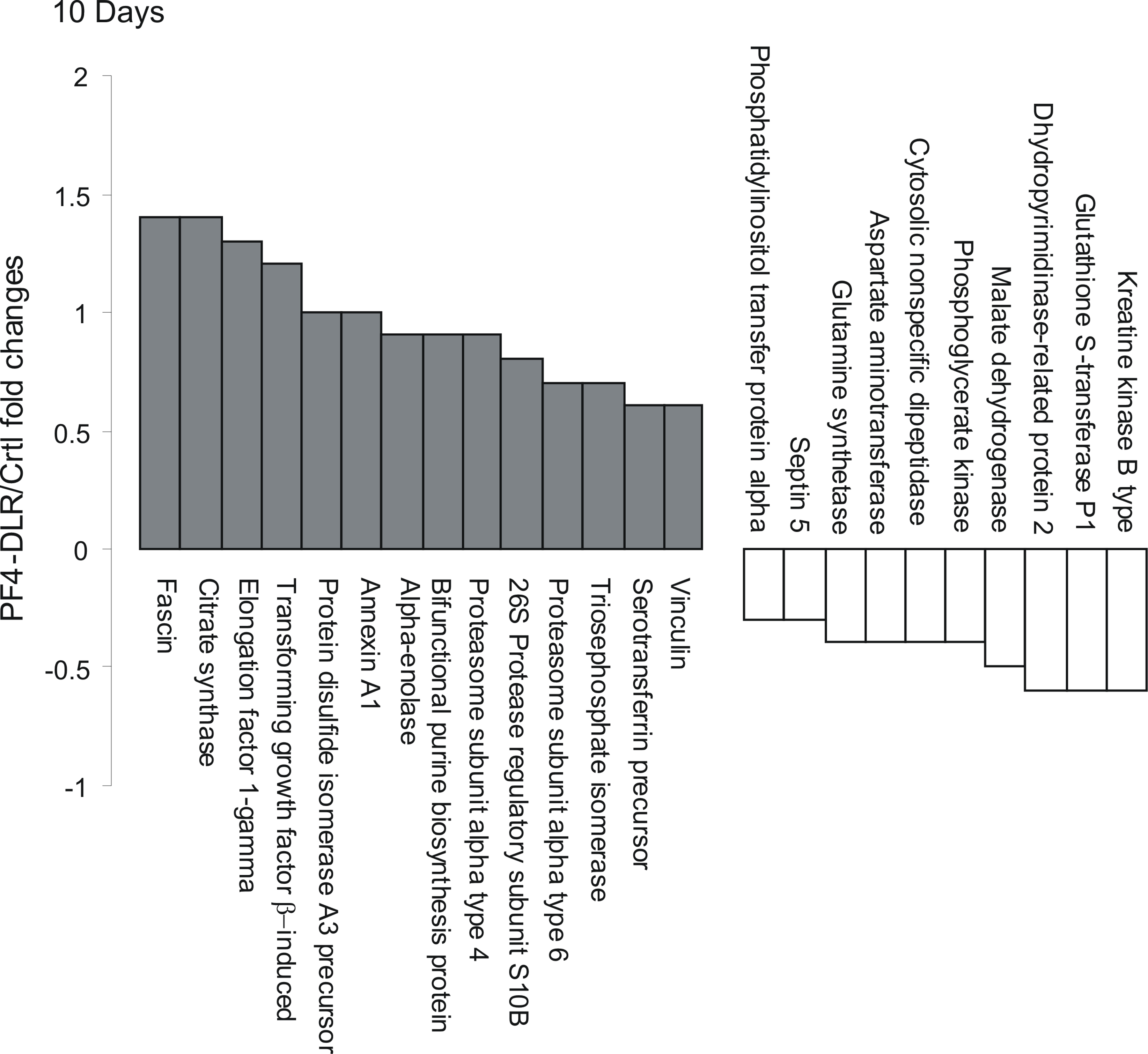

Supplement: Figure S2 — List of differentially expressed proteins identified by MALDI-TOF after two- dimensional PAGE analysis by comparing glioblastomas treated with PF4-DLR for 10 days with corresponding untreated tumors. The graph indicates the ratio PF4-DLR treated/untreated tumors (PF4-DLR/Crtl) normalized spot volume values of the identified proteins. (3.13 MB TIF) [file pone.0013710.s002.tif]

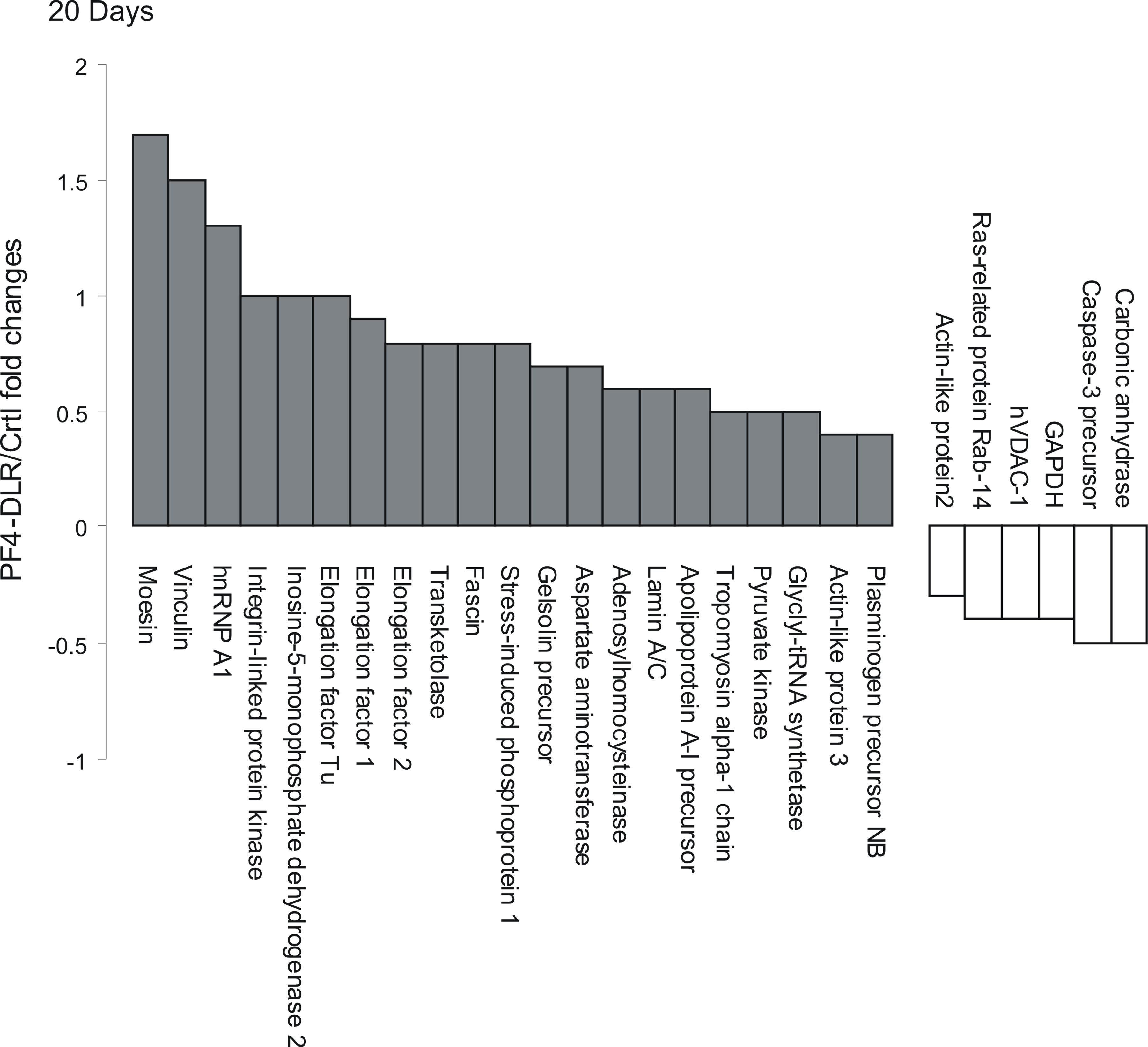

Supplement: Figure S3 — List of differentially expressed proteins identified by MALDI-TOF after two- dimensional PAGE analysis by comparing glioblastomas treated with PF4-DLR for 20 days with corresponding untreated tumors. The graph indicates the ratio PF4-DLR treated/untreated tumors (PF4-DLR/Crtl) normalized spot volume values of the identified proteins. (2.68 MB TIF) [file pone.0013710.s003.tif]
