# Supplementary material for: Anti-Angiogenic Therapy Induces Integrin-Linked Kinase 1 Up-Regulation in a Mouse Model of Glioblastoma
Source: PLoS One. 2010 Oct 29;5(10):e13710. doi: 10.1371/journal.pone.0013710 (PMC2966411; doi:10.1371/journal.pone.0013710)
Supplement: Table S1 — Proteomic analysis scheme. (0.04 MB DOC) [file pone.0013710.s004.doc]

Table S1. Proteomic analysis scheme

|  | Day 0 | Day 12 | Day 22 | Day 32 |
| --- | --- | --- | --- | --- |
| Control group | 10 mice  implanted with U87MG | 10 mice treated  with osmotic mini pumps filled with PBS | 10 mice  sacrificed |  |
| PF4-DLR  10 days | 10 mice  implanted with U87MG | 10 mice treated  with osmotic mini pumps filled with 0.5 mg of PF4-DLR | 10 mice  sacrificed |  |
| Control group | 10 mice  implanted with U87MG | 10 mice treated  with osmotic mini pumps filled with PBS |  | 10 mice  sacrificed |
| PF4-DLR  20 days | 10 mice  implanted with U87MG | 10 mice treated  with osmotic mini pumps filled with 0.5 mg of PF4-DLR |  | 10 mice  sacrificed |

Table S2. ILK1 knockdown experiment scheme

|  | Day 0 | Day 12 | Day 22 | Day 32 |
| --- | --- | --- | --- | --- |
| Control group  (10 mice) | implanted withU87MG | treated with osmotic mini pumps filled with PBS |  | 10 mice  sacrificed |
| PF4-DLR  (10 mice) | implanted withU87MG | treated with osmotic mini pumps filled with 0.5 mg of PF4-DLR |  | 10 mice  sacrificed |
| PF4-DLR  plus ILK1 SiRNA  (10 mice) | implanted withU87MG | 10 mice treated  with osmotic mini pumps filled with 0.5 mg of PF4-DLR | 10 mice treated  with osmotic mini pumps filled with 0.2 mg of ILK1 siRNA | 10 mice  sacrificed |
